# Gut microbiota composition alteration analysis and functional categorization in children with growth hormone deficiency

**DOI:** 10.3389/fped.2023.1133258

**Published:** 2023-02-24

**Authors:** Congfu Huang, Dongming Meng, Yinhu Li, Shiyang Lu, Wei Yang, Bin Wu, Shufen Chen, Zhenyu Yang, Haiying Liu

**Affiliations:** ^1^ Department of Pediatrics, Longgang Maternity and Child Institute of Shantou University Medical College (Longgang District Maternity & Child Healthcare Hospital of Shenzhen City), Shenzhen, China; ^2^ Department of Computer Science, City University of Hong Kong, Hong Kong, Hong Kong SAR, China; ^3^ Department of Pediatrics, The People’s Hospital of Shenzhen Baoan District, Shenzhen, China; ^4^ School of Statistics and Data Science, NanKai University, Tianjin, China; ^5^ Department of Pediatrics, Affiliated Shenzhen Maternity and Child Healthcare Hospital, Southern Medical University, Shenzhen, China

**Keywords:** high-throughput sequencing, growth hormone deficiency, GM, KEGG functional category, composition

## Abstract

**Objective:**

To study changes in the composition and functions of the gut microbiota (GM) in children with growth hormone deficiency (GHD) using high-throughput sequencing.

**Methods:**

Thirty-three children with GHD diagnosed in Longgang District Maternity and Child Health Hospital were included in the disease group and 24 healthy children of the same age comprised the control group. Total DNA was extracted and amplified from stool samples obtained from all subjects. High-throughput sequencing was used to analyze the GM composition and functions.

**Results:**

The GM from the two groups of children showed significant differences in α-diversity (*P* < 0.05). In comparison with the control group, the abundance of the phylum *Bacteroidetes* was significantly higher (45.96% vs. 65.71%) while the *Firmicutes* count was significantly lower (47.09% vs. 25.20%). At the genus level, the abundance of *Prevotella* in the disease group was significantly higher (3.16% vs. 20.67%) and that of *Lachnospiracea incertae sedis*, *Clostridium* XlVa, and *Megamonas* was lower (6.576% vs. 1.75%; 4.51% vs. 0.80%; 5.08% vs. 2.02%, respectively). GM functions, including those involved in membrane_transport, energy_metabolism, poorly_characterized, metabolism_of_cofactors_and_vitamins, glycan_biosynthesis_and_metabolism, transcription, folding,_sorting,_and_degradation, were significantly altered in the disease group. The abundance of various GM components was correlated with endocrine hormone levels.

**Conclusion:**

Significant alterations in the GM are seen in children with growth hormone deficiency, which may affect both energy metabolism and the levels of endocrine hormones, potentially leading to growth restriction.

## 1. Introduction

Short stature is defined as a height of less than two standard deviations or less than the third percentile among children of the same sex, age, or race. Growth hormone deficiency (GHD) is a growth disorder caused by reduced or absent production of growth hormone (GH). It is one of the most common causes of short stature in children, accounting for 38.6% of all causes (1). The worldwide incidence of GHD in children varies between 1/4,000 and 1/10,000 and most children show idiopathic GHD (2).

The stability of the gut microbiota (GM) is an important factor influencing the growth and development of children (3). Intestinal microorganisms and metabolites such as short-chain fatty acids (SCFAs) can regulate the production of hormones related to bone health, including sex steroids, vitamin D, and serotonin (4, 5). In addition, they mediate signal transduction *via* the intestinal–brain axis and affect the secretion of GH-releasing peptide, somatostatin, and leptin, all of which regulate the GH/insulin-like growth factor-1 (IGF-1) axis and modulate processes such as GH secretion, appetite regulation, and bone growth (1, 6–10). Growth hormone can not only directly promote the growth of all organs but also stimulate the production of IGF-1. The latter is an effective growth factor that plays a synergistic role with growth hormone to maintain overall growth and metabolism (11, 12). Conversely, GH or IGF-1 can also affect the composition and functions of the GM in different ways (1). Li et al. (13) reported significant changes in the GM of children with idiopathic short stature where intestinal *Clostridium* and *Eubacterium* were significantly and positively correlated with their height standard deviation score (SDS) and IGF-1 SDS. The authors believed that the decrease in IGF-1 synthesis by *Clostridium* and *Eubacterium* through SCFAs might be one of the underlying causes.

The hypothalamus–pituitary–IGF-1 axis is the main hormonal regulator of growth and development, of which GH and IGF-1 are key components (14). GHD children have reduced levels of GH and IGF-1. Imbalances in the GM can lead to endocrine hormone disorders. We speculate that children with GHD may also have GM imbalances. In this study, the intestinal composition and function of GHD children and healthy children of the same age were compared, and correlations between their GM and several hormones were analyzed to explore the characteristics of the GM of GHD children and the possible mechanism of action.

## 2. Materials and methods

### 2.1. Sample screening

We selected 33 children with GHD diagnosed at Longgang District Maternity and Child Health Hospital as the disease group, and 24 healthy children of the same age as the control group. The ages of children in the two groups ranged between 5 and 14 years, with no statistical difference seen in the comparative analysis (*P* > 0.05) (Table 1). All the children with GHD were diagnosed at the Department of Growth and Development, Shenzhen Longgang District Maternity and Child Health Hospital. The disease group met the diagnostic criteria for GHD in Chinese children (15): ① Below the third percentile of the height of normal healthy children of the same age and sex (−1.88 standard deviations [−1.88 SD] or minus 2 standard deviations [−2 SD]); ② Annual growth rate <5 cm/year; ③ Symmetrical dwarfism and childish face; ④ Normal intelligence development; ⑤ Bone age lagging behind actual age; ⑥ Peak values of two GH drug provocation tests of <10 µg/L; ⑦ Lower than normal level of serum IGF-1. The exclusion criteria for children in the two groups included: ① Severe liver or gastrointestinal disorders; ② Severe infection; ③ Treatment with antibiotics or probiotic preparations within one month before the test. All children provided informed consent from their guardians before enrollment.

**Table 1:** Comparison of clinical information between children in the disease and control groups ($\bar{x}\pm s$).

Group	Age (year)	Gender (male/female)	Weight (kg)	Height (cm)	IGF-1
Disease group (*n* = 33)	8.73 ± 2.40	21/12	22.53 ± 1.02	120.72 ± 1.87	179.71 ± 75.73
Control group (*n* = 24)	8.78 ± 2.04	14/10	27.45 ± 1.34	128.55 ± 2.49	235.55 ± 70.89
F/t Value	0.057	0.165	0.037	2.566	2.171
*P*-value	0.955	0.685	0.154	0.013	0.038

### 2.2. Sample handling and species annotation

#### 2.2.1. Collection of fecal samples for DNA extraction and sequencing from two groups of children

Approximately 5 g of the middle section of the feces was collected and immediately frozen and stored at ‒80°C. The samples were transported on dry ice to Shenzhen Micro Health Gene Technology Co., Ltd. for high-throughput sequencing. MoBio's PowerSoil® DNA Isolation Kit was used to extract bacterial DNA from fecal samples. Amplification of the V3 – V4 region of the 16S rRNA gene in DNA was performed by polymerase chain reaction (PCR). Amplified samples were sequenced using the Illumina MiSeq high-throughput sequencing platform.

#### 2.2.2. Sequencing data analysis

Low-quality reads were filtered from the sequencing data using self-programming bioinformatics tools, and the data were spliced using FLASH software (v12.11, http://ccb.jhu.edu/software/FLASH/index.shtml). The splicing sequences were aggregated into OTUs (sortable elements) with USEARCH, which were compared with the bacterial library (Greengene V201305) to obtain the GM compositions of all samples. The bacterial abundance in the samples of both groups was analyzed only at the phylum and genus levels.

### 2.3. Statistical methods

The ade4 package in R (v3.3.3) software was used to perform principal component analysis (PCA) based on the composition and relative abundance of bacteria in all samples at the genus level. The overall distribution of the microbiota compositions in the two groups was plotted. Bacteria were classified to the phylum and genus levels, and different species between the two groups were investigated by the Wilcoxon method where *P* < 0.05 indicated a significant difference. The 16S rDNA sequencing data were used to evaluate differences in bacterial functions between the two groups of children based on the functional analysis performed by the Kyoto Encyclopedia of Genes and Genomes (KEGG) database. SPSS 22.0 software was used for general data analysis. The age, weight, height, and IGF-1 values were compared by *χ*^2^ tests or two-group independent sample *t*-tests.

## 3. Results

### 3.1. Comparison of differences in the composition of the GM

**Figure 1:**
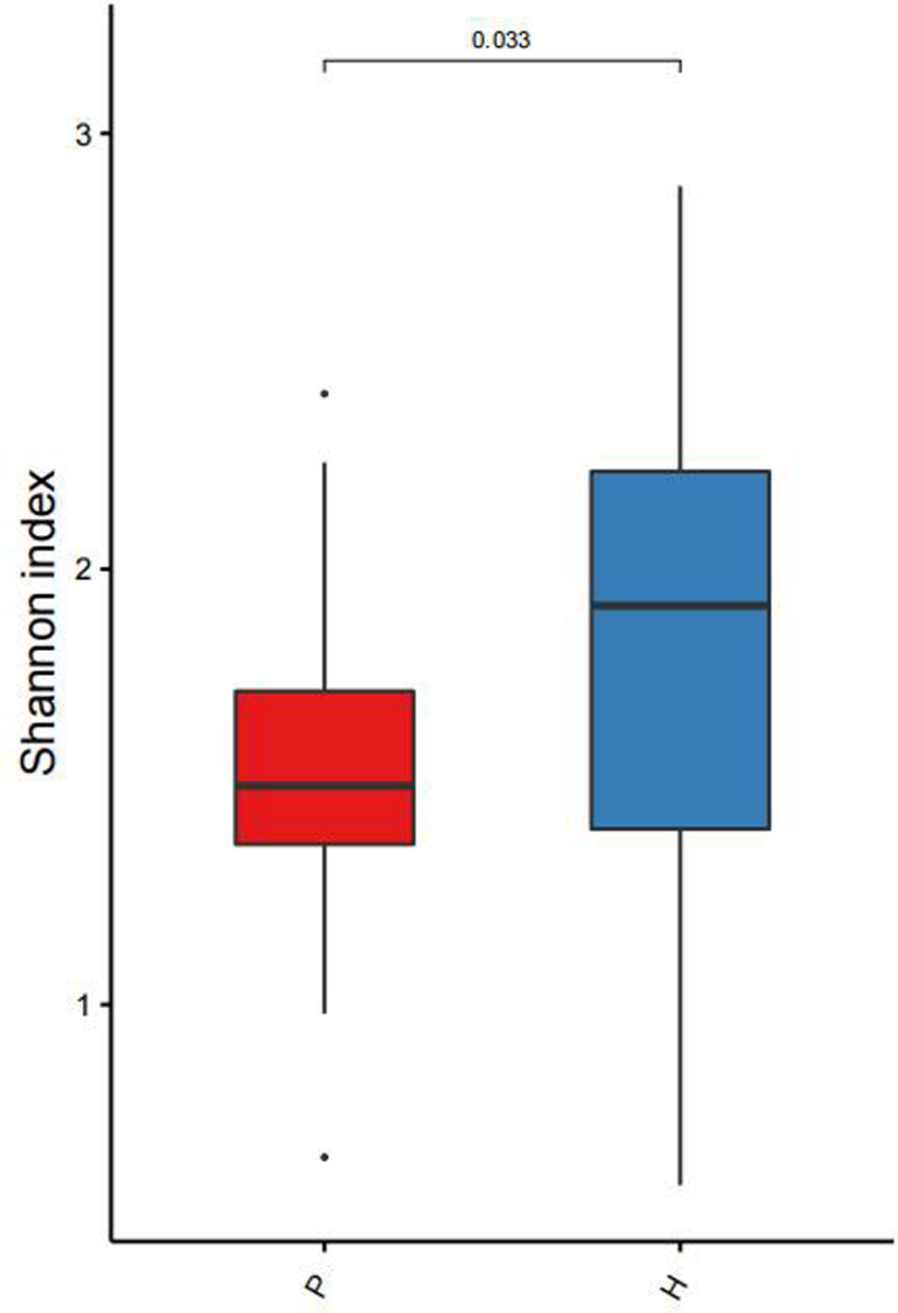
Chart showing comparison of microbiota diversity between the two groups.

**Figure 2:**
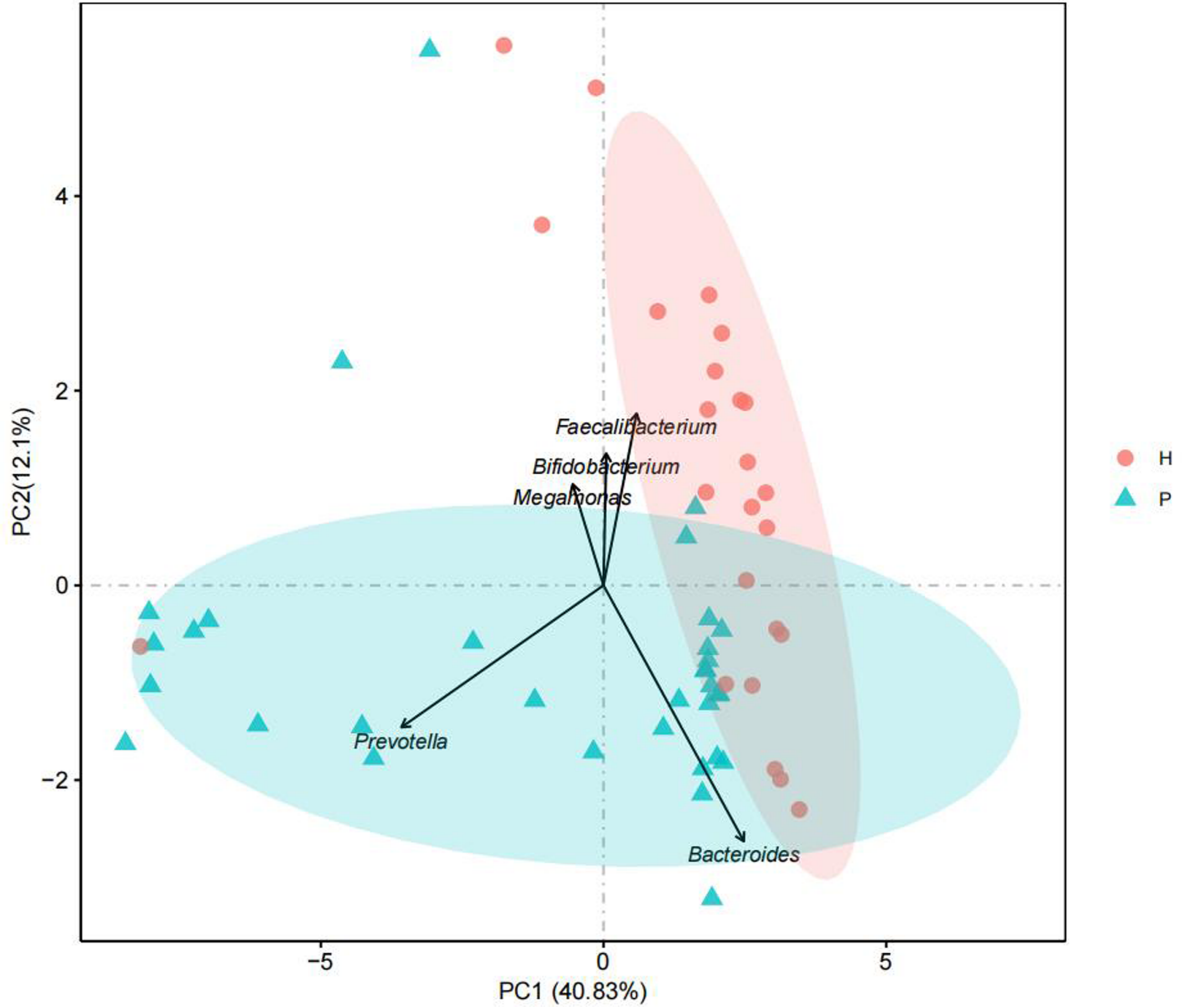
Principal component analysis.

The GM of two groups of children showed significant differences in α-diversity (*P* = 0.033) (Figure 1). We used PCA to reduce the dimensionality of the GM data of the two groups, finding that there were marked differences in the GM between the two groups. The genera that contributed most to this difference included *Prevotella* (*P* < 0.001), *Megamonas* (*P* = 0.01), *Bacteroides* (*P* = 0.765), *Bifidobacterium* (*P* = 0.011), and *Faecalibacterium* (*P* = 0.094) (Figure 2).

### 3.2. Comparison of the dominant bacterial phyla between the two groups of children

**Figure 3:**
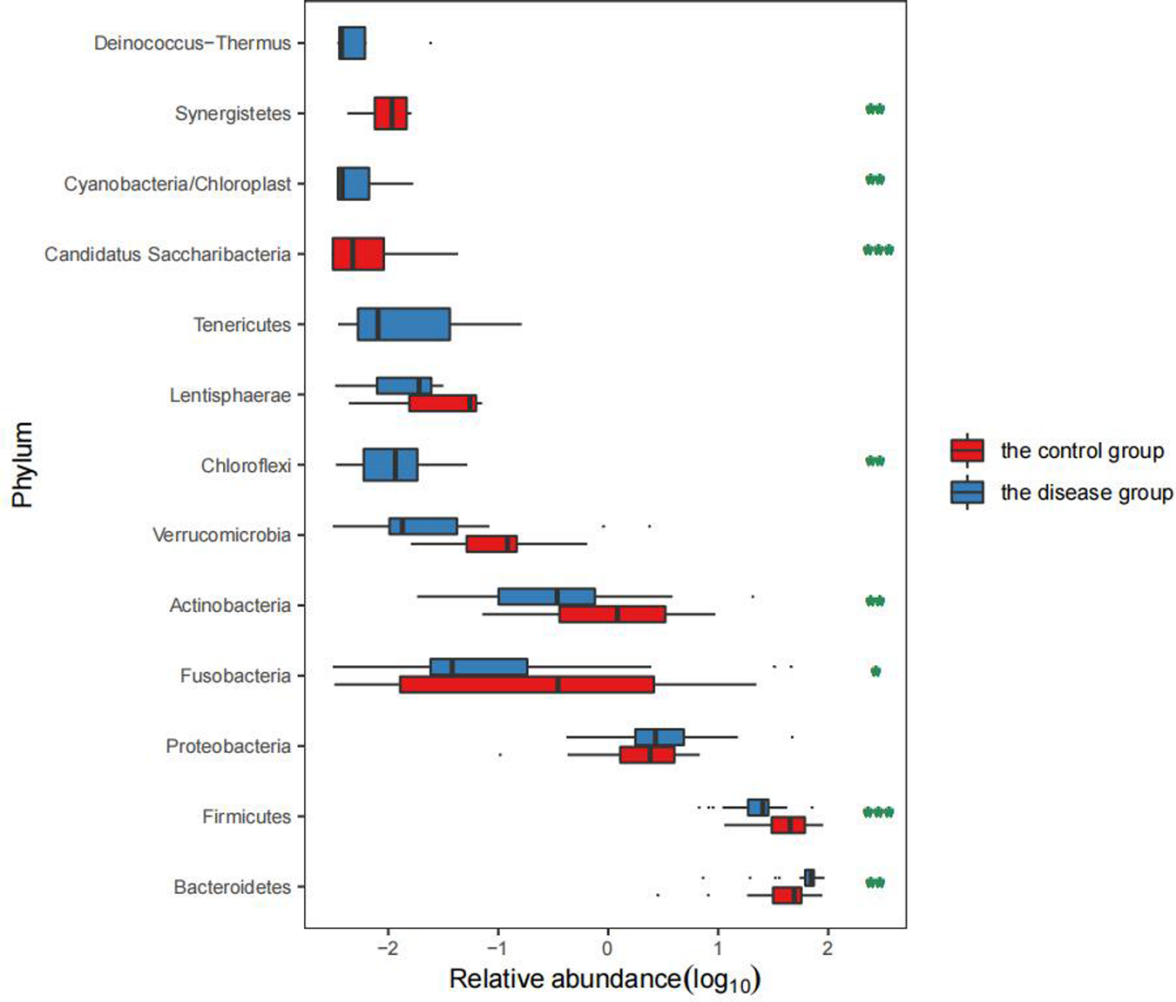
Comparison of the GM levels in the two groups.

The top five dominant bacterial phyla differed between the groups with a significant increase in the abundance of *Bacteroides* in the disease group (*P* = 0.000) together with a significant reduction in the abundance of *Firmicutes* (*P* = 0.000). In addition, there was also a significant difference between the two groups in the abundance of *Fusobacteria* and *Actinomycetes* (*P* < 0.05) (Table 2 and Figure 3).

**Table 2:** Analysis of dominant bacteria in children in the disease and control groups (top 5).

Top 5 dominant phyla	Disease group		Control group		*P*-value	FDR
	Mean (%)	SD (%)	Mean (%)	SD (%)		
*Bacteroidetes*	65.71	18.02	45.96	21.21	0.000	0.002
*Firmicutes*	25.20	12.11	47.09	20.66	0.000	0.000
*Proteobacteria*	4.90	8.28	2.80	1.86	0.312	0.369
*Fusobacteria*	2.71	9.86	1.54	4.69	0.011	0.018
*Actinobacteria*	1.14	3.64	2.54	2.98	0.002	0.004

### 3.3. Comparison of the dominant bacterial genera between the two groups of children

**Figure 4:**
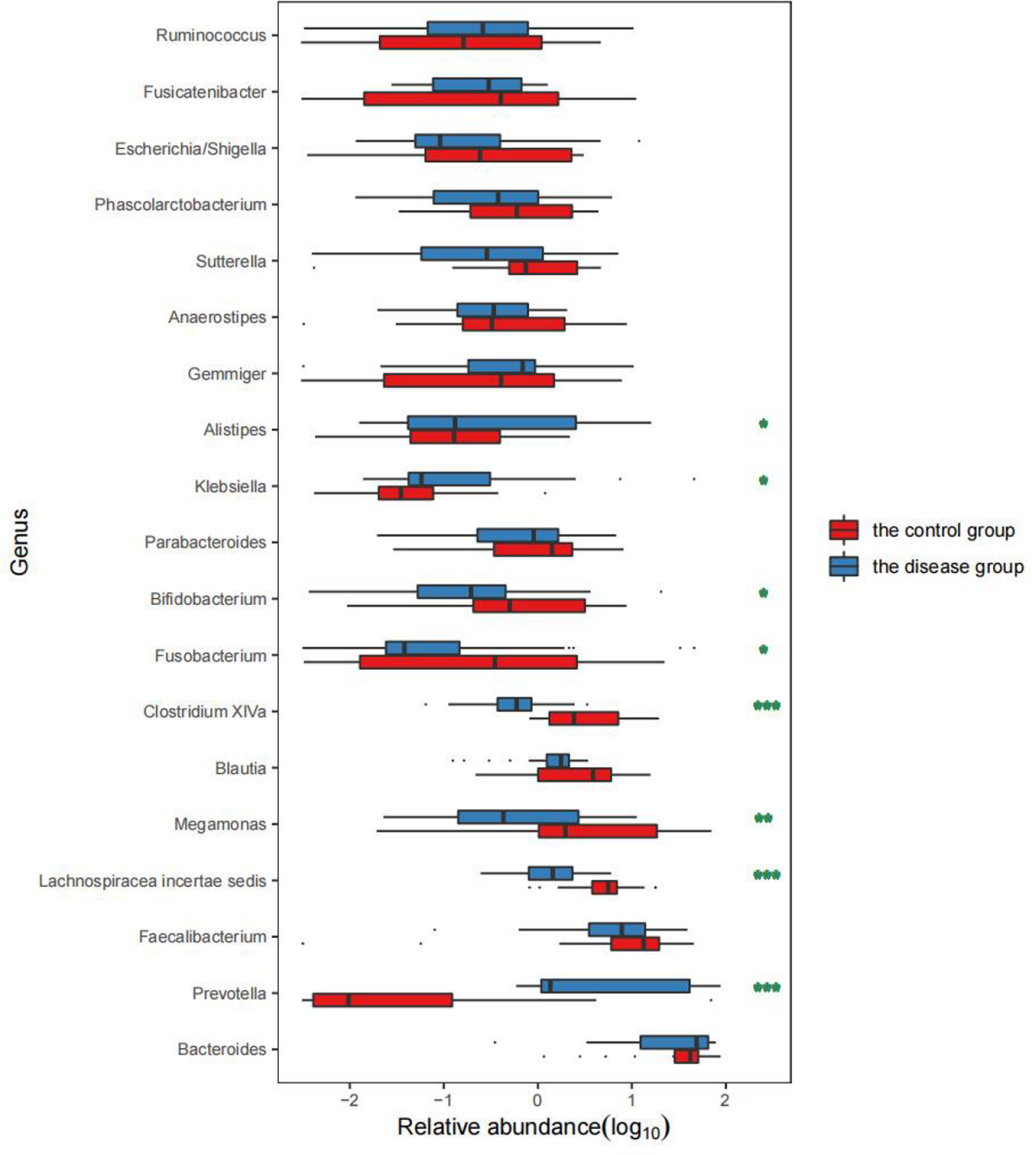
Comparison of the abundance of bacterial genera between the two groups. Remarks: * indicates *P* < 0.05, ** indicates *P* < 0.01, and *** indicates *P* < 0.001 statistically significant differences between the two groups. The higher the number of asterisks, more significant the difference.

We selected the top 15 dominant bacterial genera in the two groups for comparison. The results showed that the abundance of *Prevotella*, *Fusobacterium*, *Klebsiella*, and *Alistipes* was significantly increased in the disease group (*P* < 0.05) while that of *Lachnospiracea incertae sedis*, *Megamonas*, *Blautia*, *Clostridium* XlVa, *Bifidobacterium,* and *Eubacterium* was significantly decreased (*P* < 0.05) (Table 3 and Figure 4).

**Table 3:** Comparison of the top 15 dominant bacterial genera in the two groups of children.

Top 15 dominant genus	Disease group		Control group		*P*-value	FDR
	Mean (%)	SD (%)	Mean (%)	SD (%)		
*Prevotella*	20.67	29.63	3.16	14.46	0.000	0.000
*Lachnospiracea incertae sedis*	1.75	1.34	6.57	4.70	0.000	0.000
*Megamonas*	2.019	2.85	5.08	15.17	0.001	0.008
*Blautia*	1.69	0.82	4.05	3.59	0.027	0.077
*Clostridium* XlVa	0.80	0.67	4.51	4.32	0.000	0.000
*Fusobacterium*	2.71	9.86	1.54	4.69	0.012	0.041
*Bifidobacterium*	1.03	3.64	2.17	2.89	0.011	0.040
*Klebsiella*	1.89	8.21	0.10	0.25	0.004	0.017
*Alistipes*	1.62	2.95	0.28	0.55	0.006	0.022
*Gemmiger*	1.00	1.82	0.90	1.79	0.031	0.084
*Roseburia*	0.63	0.81	0.61	1.22	0.018	0.052
*Ruminococcus2*	0.41	0.60	0.79	0.79	0.022	0.064
*Streptococcus*	0.18	0.31	0.75	1.14	0.043	0.098
*Oscillibacter*	0.42	0.53	0.20	0.29	0.041	0.098
*Eubacterium*	0.02	0.04	0.58	2.71	0.046	0.103

### 3.4. Alterations of GM functions in the GHD children

**Figure 5:**
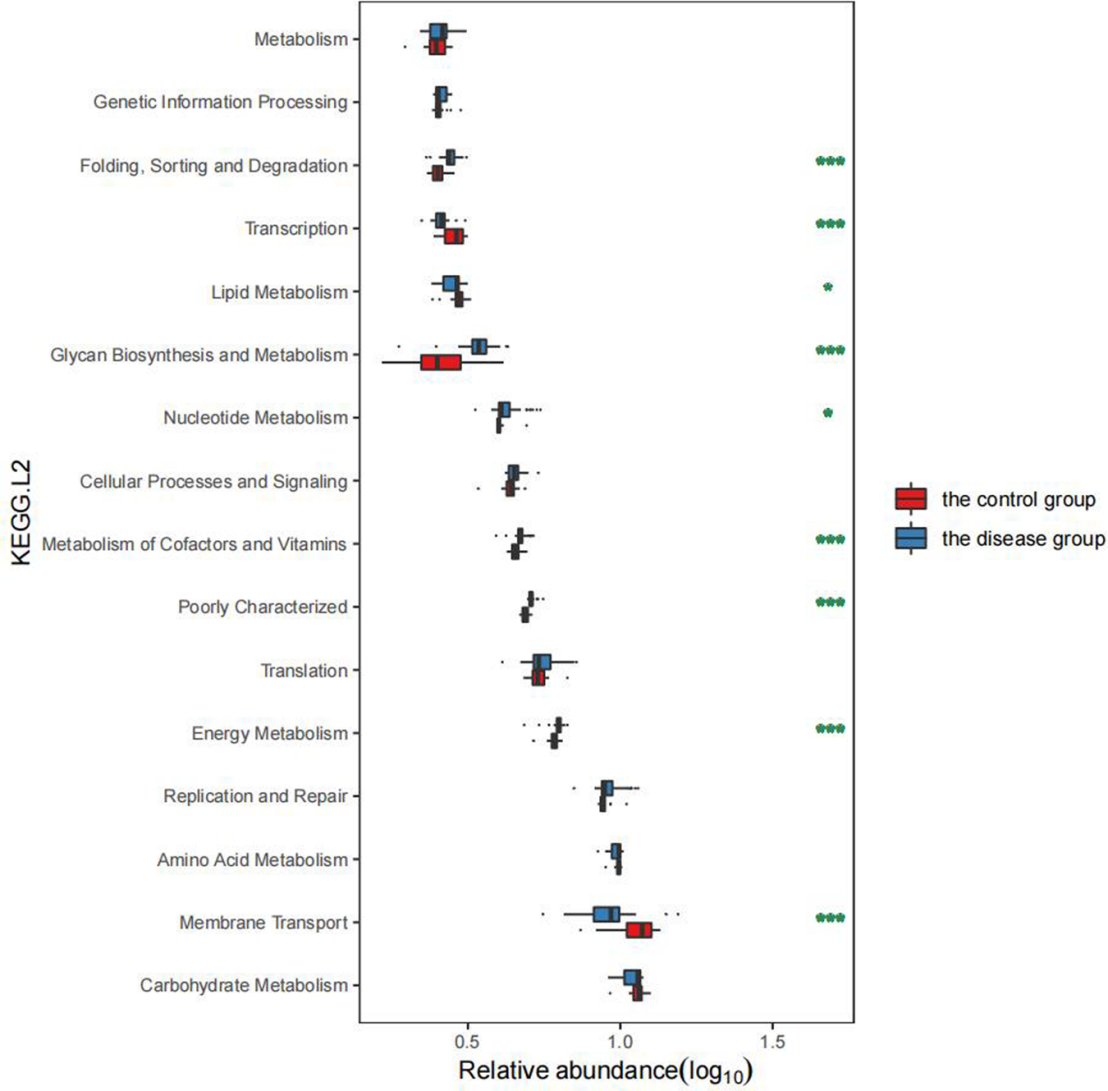
Comparison of GM functions between the two groups of children.

In comparison with the healthy children, the GHD patients showed significant changes in GM functions, including the decreased “Membrane transport” (*P* < 0.001, FDR < 0.001), “Lipid metabolism” (*P* = 0.025, FDR = 0.042), and “Transcription” (*P* < 0.001, FDR < 0.001, Figure 5), which indicated the. In contrast, the functional categories, such as “Energy metabolism” (*P* < 0.001, FDR < 0.001), “Metabolism of cofactors and vitamins” (*P* < 0.001, FDR < 0.001), “Nucleotide metabolism” (*P* = 0.008, FDR = 0.016), “Glycan biosynthesis and metabolism” (*P* < 0.001, FDR < 0.001), and “Folding sorting and degradation” (*P* < 0.001, FDR < 0.001) were enriched in the GHD patients (Figure 5). These elevated GM metabolic activities in the GHD patients, especially the “Glycan biosynthesis and metabolism” function, affect the neuro-regulations in hosts and is probably related to the occurrence of GHD.

### 3.5. GM and clinical phenotypes

**Figure 6:**
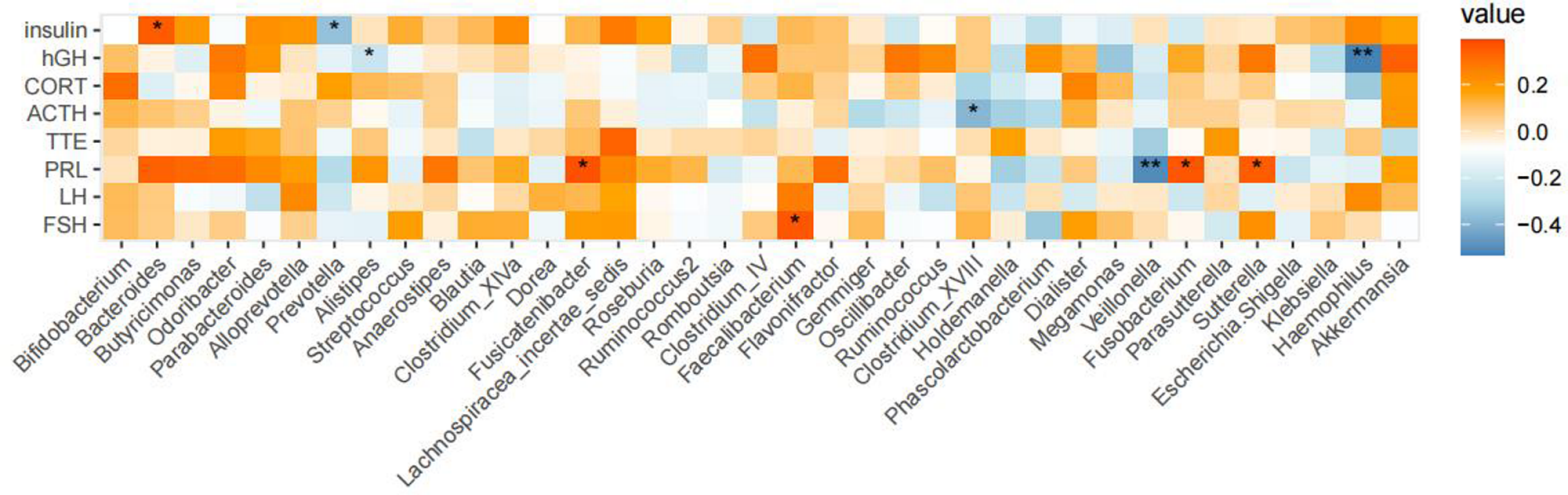
Correlations between GM compositions and endocrine hormones in children with GHD. Legend description: A correlation analysis was performed with eight clinical phenotypes and genera with a relative abundance of ≥0.1%. Results are shown as above where significance was expressed as *P < 0.05 and **P < 0.01.

Spearman's correlation analysis was used to investigate associations between the GM of children with GHD and eight endocrine hormones. Our results showed that *Bacteroides* were positively correlated and *Prevotella* was negatively correlated with insulin, while *Alistipes* and *Haemophilus* showed a negative correlation with GH. A positive correlation was also reported between *Fusicatenibacter*, *Fusobacterium,* and *Sutterella*, whereas *Veillonella* was negatively correlated with prolactin. *Faecaliterium* and FSH were positively correlated (Figure 6).

## 4. Discussion

### 4.1. The GM composition differed markedly between the disease and control groups

Compared with the healthy controls, children in the disease group showed reduced α-diversity in the GM, consistent with results reported in malnourished children (16). The phylum *Bacteroides* was more abundant in children from the disease group than in those from the control group while the opposite trend was observed for *Firmicutes*, in contrast to findings on obese and diabetic patients (17). The abundance of *Prevotella* in the disease group was also significantly higher than that in the control group. *Prevotella* can degrade broad-spectrum plant polysaccharides (18), and carbohydrate-based diets tend to form a *Prevotella*-dominated “gut type.” Increased abundance of *Prevotella* abundance has been shown to reduce blood sugar and insulin levels, thus affecting energy absorption and promoting weight loss (19). In the disease group, the abundance of *Fusobacterium*, *Klebsiella*, *Alistipes*, and other genera was found to be significantly increased. *Fusobacterium* is present in the normal oral flora and can inhibit the immune response as well as promote the transformation of inflammation to malignancy (20). An increase in the abundance of both *Klebsiella* and *Alistipes* has been shown to be associated with intestinal inflammation (21, 22); therefore, the increase in the population of these genera can promote chronic inflammation in the intestine and disrupt the function of the intestinal barrier. This can lead to a cellular biochemical imbalance, reduced absorption capacity, and increased susceptibility to enteric pathogen infections, and consequently affect energy metabolism and nutrient absorption (23). In addition, *Klebsiella* and *Alistipes* are both associated with neurological diseases (24) and can produce neurotransmitter-related metabolites such as serotonin, dopamine, and histamine (25). These neurotransmitters enter the brain through the gut-brain axis to regulate the energy balance and function of the hypothalamus (26, 27). The hypothalamus is the highest regulatory center of thehypothalamic–pituitary–growth axis (HPA) and can reduce appetite and cause weight loss (28, 29). *Lachnospiracea incertae sedis*, *Megamonas*, *Blautia*, *Clostridium* XlVa, and *Bifidobacterium* were found to be significantly reduced in the intestines of the disease group, which could lead to reduced concentrations of SCFAs such as butyric acid produced by these beneficial bacteria (30). Decreased abundance of *Lachnospiracea incertae sedis* might also affect protein synthesis (31), disturb the intestinal energy supply, and retard growth and development. Jensen et al. (5) reported that increased *Prevotella* abundance together with reduced numbers of *Bifidobacterium* can reduce the levels of growth hormone-releasing peptide (GHRP) and leptin, thus reducing the release of GH.

### 4.2. Significant differences in GM function between the two groups

The enriched functional categories in the GHD group included “Replication and repair, Energy metabolism, Poorly characterized, Metabolism of cofactors and vitamins, Nucleotide metabolism, Cellular processes and signaling, Nucleotide metabolism, Glycan biosynthesis and metabolism, Transcription, Folding sorting and degradation”. Children with GHD showed dysregulation in energy metabolism, vitamin and related factor metabolism, and polysaccharide metabolism and biosynthesis. Considering that *Prevotella* significantly increases the catabolism of carbohydrates and that the abundance of butyric acid and other bacteria such as *Lachnospiracea incertae sedis* is significantly reduced in children with GHD, the GM imbalance in this population may affect the functions of the flora. This phenomenon may lead to chronic inflammation of the intestine and poor intake and absorption of nutrients such as fats and proteins, affecting both growth and development.

### 4.3. GM is closely related to the clinical phenotype

We conducted a correlation analysis of the GM and endocrine hormones and found that *Prevotella* abundance was negatively correlated with insulin. Significantly higher abundance of *Prevotella* can affect insulin secretion, which can not only regulate food intake (19, 32) but also modulate blood glucose levels through signaling pathways essential for maintaining energy storage, glucose metabolism, sugar production, adipogenesis, cell growth, survival, and reproduction (33). We speculate that this significant increase in *Prevotella* abundance may be detrimental to growth and development. We also found a variety of other intestinal bacteria related to endocrine hormones and confirmed the interaction between the GM and endocrine hormones. Maintaining the stability of the GM is conducive to the promotion of growth and development.

## 5. Conclusion

There was a significant reduction in the α-diversity of the intestinal microbial composition in GHD children, together with an increased abundance of *Bacteroides* and reduced numbers of *Firmicutes*. *Fusobacterium*, *Klebsiella*, *Alistipes*, and other genera were significantly enriched in children with GHD while the numbers of *Lachnospiracea incertae sedis*, *Megamonas*, *Blautia*, *Clostridium* XlVa, and *Bifidobacterium* were significantly reduced. These imbalances in the GM were predicted to affect pathways involved in energy metabolism and biosynthesis, as well as induce abnormal secretion of insulin and other endocrine hormones, which may promote the occurrence and development of GHD.

### 5.1. Deficiencies and next steps

There are many factors that cause insufficiency in GH secretion in children with GHD, and GM imbalance may be one of the major factors. On the one hand, GM imbalance leads to the abnormal secretion of endocrine hormones as well as an abnormal production of microbial metabolites, especially neurotransmitters, that can influence the HPA through the gut–brain axis. The sample size in the present study was small, consisting of only 33 children with GHD; hence, large-sample, multi-center research is needed to verify the associations between the GM and GHD. Studies combined with metabolomics could better clarify the mechanism of action of the GM and its metabolites in growth and development.

## Data Availability

The data presented in the study are deposited in the NCBI sequence Archive (SRA) database, accession number: PRJNA899674 .The data can be found at the following link: https://dataview.ncbi.nlm.nih. gov/object/PRJNA899674?reviewer=tu7mnej3p04c61u31c6f4v5hgo.
